# Provider perceptions of challenges to identifying women Veterans with hazardous substance use

**DOI:** 10.1186/s12913-022-07640-z

**Published:** 2022-03-04

**Authors:** Karleen F. Giannitrapani, Jesse R. Holliday, Andrew W. Dawson, Alexis K. Huynh, Alison B. Hamilton, Christine Timko, Katherine J. Hoggatt

**Affiliations:** 1 Center for Innovation to Implementation (Ci2i), VA Palo Alto Health Care System, Palo Alto, CA United States of America; 2 Department of Primary Care and Population Health, School of Medicine, Stanford University, Palo Alto, CA United States of America; 3 Mind and Society Center, Dornsife College of Letters, Arts and Sciences, University of Southern California, Los Angeles, CA United States of America; 4 Center for the Study of Healthcare Innovation, Implementation & Policy, VA Greater Los Angeles Healthcare System, Los Angeles, CA United States of America; 5 Department of Psychiatry and Behavioral Science, David Geffen School of Medicine, University of California Los Angeles, Los Angeles, CA United States of America; 6 Department of Psychiatry and Behavioral Sciences, School of Medicine, Stanford University, Palo Alto, CA United States of America; 7 San Francisco VA Health Care System, San Francisco, CA United States of America; 8 Department of Medicine, University of California San Francisco, San Francisco, CA United States of America

**Keywords:** Women veterans, Hazardous substance use, Identification

## Abstract

**Background:**

Approximately one-third of women Veterans Health Administration (VHA) users have substance use disorders (SUD). Early identification of hazardous substance use in this population is critical for the prevention and treatment of SUD. We aimed to understand challenges to identifying women Veterans with hazardous substance use to improve future referral, evaluation, and treatment efforts.

**Methods:**

Design: We conducted a secondary analysis of semi-structured interviews conducted with VHA interdisciplinary women’s SUD providers at VA Greater Los Angeles Healthcare System. Participants: Using purposive and snowball sampling we interviewed 17 VHA providers from psychology, social work, women’s health, primary care, and psychiatry. Approach: Our analytic approach was content analysis of provider perceptions of identifying hazardous substance use in women Veterans.

**Results:**

Providers noted limitations across an array of existing identification methodologies employed to identify women with hazardous substance use and believed these limitations were abated through trusting provider-patient communication. Providers emphasized the need to have a process in place to respond to hazardous use when identified. Provider level factors, including provider bias, and patient level factors such as how they self-identify, may impact identification of women Veterans with hazardous substance use. Tailoring language to be sensitive to patient identity may help with identification in women Veterans with hazardous substance use or SUD who are not getting care in VHA but are eligible as well as those who are not eligible for care in VHA.

**Conclusions:**

To overcome limitations of existing screening tools and processes of identifying and referring women Veterans with hazardous substance use to appropriate care, future efforts should focus on minimizing provider bias, building trust in patient-provider relationships, and accommodating patient identities.

## Background

Historically, medical schools have not emphasized training in addiction-related medicine, resulting in a healthcare workforce largely under prepared and under qualified to identify patients with hazardous substance use or substance use disorder (SUD) [1–4]. Despite the United States experiencing the highest annual overdose death toll in the nation’s history in 2020, [5] the majority of medical schools dedicate a mere handful of hours to teaching addiction medicine, which is marginal in comparison to the time spent educating students on other chronic conditions [3]. This, paired with the confusing and problematic diagnostic language that renders many providers unable to distinguish SUD from substance dependence from hazardous substance use, [3] may lead providers to mis- or under-identify substance use concerns or disorders in their patient populations.

Identifying the target population for appropriate treatment is a ubiquitous challenge healthcare systems face [6]. Across many conditions, providers and systems strive to capture at-risk individuals while minimizing incorrect identification (i.e., false positives). Identifying patients with specific conditions, however, can be particularly challenging when a diagnosis may also come with negative consequences, or perceived negative consequences for patients, as in the case of SUD, characterized by symptoms including the diminished ability to control the use of alcohol or other drugs regardless of associated harmful consequences [7]. Existing policies across the United States give women reason to fear criminal justice consequences of disclosing hazardous substance use, specifically around pregnancy and motherhood [8]. In many states, hazardous substance use during pregnancy is considered child abuse under the law and can result in legal consequences for women (i.e., civil commitment) [8]. For the purposes of this paper, hazardous substance use refers to repeatedly using substances in ways that increase the risk of causing harm and may lead to negative consequences, to a degree that necessitates provider attention [7].

SUD is a debilitating chronic health condition that negatively impacts quality of life and health [9]. SUD is highly correlated with multiple comorbid physical and mental health conditions, with a strong association between the severity of SUD and the magnitude of comorbidity [10]. Women are disproportionately impacted by the negative consequences of hazardous substance use and SUD [11]. Annual diagnosis rates for SUD are increasing for both men and women [12]. Among Veterans, studies show a significantly higher rate of increase in SUD diagnoses for women than men [12]. Because women Veterans have a high prevalence of comorbid mental and physical health conditions, they comprise a high-risk, potentially high-need population that may need and benefit from tailored specialty SUD services [12].

Women Veterans with hazardous substance use and SUD can have complex care needs. First, they are more likely to have a history of trauma than men Veterans as well as women non-Veterans [13]. For example, many women Veterans experience military sexual trauma which is associated with both mental health disorders and hazardous substance use [14]. Women Veterans with SUD may also be up to ten times more likely to suffer from post-traumatic stress disorder (PTSD) than their counterparts without SUD [15]. Second, women Veterans with SUD have higher mortality rates, and SUD increases the likelihood of suicide, particularly in the presence of comorbid mental health disorders [16].

How women self-identify around complex and sensitive issues such as hazardous substance use and Veteran status may vary based on context and impact their ability to obtain or access referral to resources for SUD diagnosis and treatment through the VHA. As such, it is important to consider the ways in which identity and self-identification impact how women Veterans with hazardous substance use or diagnosed SUD access and interact with the healthcare system [17].

Early identification that leads to early treatment may prevent more severe consequences of hazardous substance use and SUD for women [18]. Several factors indicate early identification is especially critical for women with hazardous substance use including that: (a) by the time women reach SUD diagnosis and treatment, they have higher addiction severity than men as well as more health problems, both physical and psychological, [19] and (b) because women experience the well documented telescoping phenomenon, meaning they may progress from hazardous substance use to diagnosable SUD more rapidly than men, there is a significantly shorter timeframe for early detection and intervention.[20].

Research has demonstrated a need for improvement in screening and referral techniques for women Veterans with hazardous substance use for decades [21]. For example, widely used screening tools, including the Alcohol Use Disorders Identification Test (AUDIT-C) -- a questionnaire that is considered the standard to reliably diagnose alcohol in the general populations–suffer from low sensitivity if not tailored for women [22–24]. In order to improve case-finding, new screening tools are being developed and deployed [25]. However, currently, there is a gap in the literature with regard to a gold standard method to identify women Veterans with hazardous substance use, inside and outside of the VHA. We aimed to understand how women with hazardous substance use are currently identified by VHA providers, challenges to identifying women Veterans with hazardous substance use, and how to think about improving future identification efforts.

## Methods

### Design

 All data management and analysis were conducted using qualitative analytic software, ATLAS.ti (v. 7). We conducted a secondary analysis of semi-structured interviews with key informants at three distinct VA Greater Los Angeles Healthcare System sites. These sites included one large medical center, one urban community-based outpatient clinic, and one suburban community-based outpatient clinic. We interviewed purposively sampled interdisciplinary women’s health providers to understand how they identify women Veterans with hazardous substance use and identification barriers. Oral consent was obtained from all participants and the study was approved by the Greater Los Angeles institutional review board.

### Interviews

Identification and recruitment of multidisciplinary provider key informants was done using a purposive snowball sampling approach [26, 27]. We recruited key informants from disciplines including psychology, social work, women’s health, primary care, and psychiatry. Providers had to currently work with women Veterans to be considered eligible. Seventeen interdisciplinary providers agreed to be interviewed in 2014. All interviews were completed in-person and were conducted by investigators educated in qualitative interviewing techniques (KG, ABH, AKH). Sixteen of the 17 interviews were digitally recorded, transcribed by professionals, and all identifying information was removed. For the single case (1 out of 17) where the key informant requested to not be recorded, a second investigator handwrote detailed notes while the first investigator conducted the interview.

We used a semi-structured interview guide to elucidate provider experiences with screening, identifying, referring, and treating women Veterans with SUD and hazardous substance use [28]. Probes relating to screening included: screening tools and methods used in VHA (e.g. AUDIT-C), limitations of the existing tools, challenges to identification method and processes, current referral pathways, and challenges moving patients from identification to treatment.

### Data Analysis

Analysis relied on both constant comparison and content analysis methods. Initially, two investigators (KG and AKH) independently free-coded two distinct provider interviews and each created a draft code list. The entire team of investigators combined the two draft code lists into one final code list using discussion and consensus. The team used an iterative process to develop definitions for the codes and then, by consensus, finalized the code list. Two investigators used the final code list to double-code two interviews and then, together, compared their coding practices and reconciled distinctions between the two transcripts that had been double-coded. We split the remaining transcripts in half and divided them between the aforementioned two investigators. All transcripts were either double-coded or initially coded by the primary coder and then reviewed by a separate investigator. The codes from this initial list can be conceptualized as high level “top-codes,” only one of which was used for this analysis: Identification. Next, two investigators (KG and JH) used the text output from the identification code for the content analysis for this study.

## Results

We found five key themes across the key informant interviews: 1) There are many limitations to the existing tools and methods currently employed to identify women Veterans with hazardous substance use; 2) Identification needs to be followed-up with referral to appropriate providers and resources; 3) Provider-level factors are associated with multiple challenges to identifying Veterans with hazardous substance use who would be eligible to receive care in the VHA; 4) Patient-level factors also pose challenges; 5)  Tailoring language to be sensitive to patient identity and to help avoid stigma may help with hazardous substance use identification in women with military service histories and women with hazardous substance use who are not getting care in VHA but are eligible as well as those who are not eligible for care in VHA.

### Theme 1: Providers Highlight Limitations of Current Identification Methodologies

Providers noted limitations across an array of existing identification methodologies including screening questionnaires (like the AUDIT-C) and patient-provider dialogue. Providers perceived strong patient-provider relationships as foundational to facilitating open and transparent communication and hypothesized that tools deployed outside of trusting patient-provider relationships may not elicit truthful responses given the stigma associated with a SUD diagnosis. Lack of patient trust was raised as a barrier to identifying hazardous substance use. Providers believed that patients’ fear of negative repercussions and stigma associated with having a SUD diagnosis may disincentivize full transparency about the true extent of their substance use. Finally, providers conveyed that the implementation of the identification methodologies (e.g., frequency of assessments and follow up) and the narrow focus of the tools themselves (e.g., focus on use of individual substances such as alcohol is not inclusive enough to identify hazardous use of prescriptions or illicit substances) present limitations to adequately identify the full range of hazardous substance use.

Providers expressed both empathy for their women patients and lack of clarity around who to refer for further assessment of hazardous substance use and potential SUD diagnosis. One provider demonstrated their own struggle of navigating defining the threshold between recreational substance use and hazardous substance use or diagnosable substance use disorder: *“…it gets a little tricky like determining where’s the line of is this a really serious addiction that we’re dealing with?”* “Self-referral” was emphasized as an important identification pathway.

Providers challenged some of the existing VHA policies and asserted that better identification will be difficult to achieve if there are any *“negative repercussions that happen to Veterans if they do bring it [hazardous substance use] up to a provider.”* They also highlighted that some women Veterans may still be reservists and there are many real potential negative consequences (e.g., probation or disqualification from certain positions) to such Veterans if they are honest about hazardous substance use: *“…if women aren’t feeling like they can talk about it…If they’re active duty or even like in the Reserves. I mean, yeah, there are those considerations.”*

Existing substance use and SUD screening tools may be insufficient: “*The AUDIT-C is like two questions, so not very thorough*.” The fact that healthcare intake questionnaires are not repeated limits opportunity to identify emergent hazardous substance use:

Well, actually, when I first meet the patient, I’ll ask them. “Do you drink? Do you do this?” I mean, do you have any history of any illicit drug use? How much do you…” So, I ask them specific questions… But if something has changed from the first time I’d met them, or I haven’t seen them in a while. I usually don’t re-ask some of those questions.

Screening tools outside the context of a trusting provider-patient relationship may prevent the possibility of identifying women Veterans with hazardous substance use given the associated stigma and potential negative consequences:

*“Well, we’re supposed to do the clinical reminders. I do the clinical reminders. But, I don’t know whether they [woman Veterans] would be so honest. I think it’s about really having the connection with the provider…with women, it’s the relationship…the women’s clinic is better in that, and better able to identify….But that’s really about the only way, unless somebody is really just that down and out and presents [their hazardous substance use].“*

### Theme 2: Providers emphasize the need to have a process in place to respond to hazardous use when identified

In response to questions regarding how to improve screening and identification, providers emphasized that it is critical to have processes and resources in place to meet identified needs. Referral pathways must be established before increased cases are identified with increased volume of screening:

If we then start asking the questions [substance use screening], then, we’d better pull together the list of resources, right? In some ways I think that it could be just as simple as that. Let’s pull together a list of the existing resources, be sure that all the providers have those literally at their fingertips.

Improving identification is not just about the measures themselves, it is about having an easy method to bridge from identification of hazardous substance use to referral for potential SUD diagnosis and treatment. One provider suggested what if:

...they just have to do one little click and up that list comes for them and then they could print that out and hand it to the Veteran or maybe it’s internally and they would then know which one [clinic] they wanted to make the referral to. However we actually construct the technology of it, I think sometimes it’s as simple as being sure that they’ve [primary care providers] got the list of resources, internal and external resources [e.g. places to refer patients with hazardous substance use].

Providers further emphasized that primary care based screening, while helpful for starting the process, needs to be followed by much more comprehensive assessment. A specialty SUD psychiatrist explained the value of screening as less about getting to a diagnosis, and more about having a prompt to link patients with escalating hazardous substance use from primary care to specialty providers with the capacity to evaluate, diagnose and treat a substance use disorder if needed.

“*through our standard intake assessment process, a lot of times it’s stated in the referral that they have substance use issues or we can see their audit score, that sort of thing. So a lot of times the primary care providers say, here in this clinic, if they refer them to us we’ll give them the heads-up about that, but then we definitely do a more thorough assessment …for substance use, including current use, past use, any treatment history.”*

### Theme 3: How provider perceptions and biases may impact identification of women Veterans with hazardous substance use

Providers highlighted many factors impairing their ability to adequately screen and identify indicators of hazardous substance use and SUD in women Veterans. Low prevalence of SUD and low exposure to patients with hazardous substance use or SUD was consistently noted by providers; as one provider stated, *“To be honest, I have not really, especially in recent months, have that many women Veterans come in, referrals where substance use is the primary issue.*” Another provider believed low prevalence of SUD among women Veterans to be implausible, considering the prevalence of trauma in this patient population and the comorbidity of trauma and hazardous substance use:

*"That substance abuse has not been more at the forefront of care for women Veterans considering the trauma and that some VAs, some providers said, ‘Well, first of all, we don’t see that much substance abuse among women,’ which I still have a really hard time reconciling…."*

Providers may also underestimate the impact of hazardous substance use on the patient: *“They [Providers]…think that the women who are using are not abusers.”* Finally, it was evident that providers may not consider high functioning patients with hazardous substance use to qualify for SUD referrals. Providers may overemphasize substance use as a possibly legitimate coping strategy for women Veterans instead of viewing their substance use as hazardous.

*"…at least about alcohol use, okay, so they [Providers] pick up some of that. But…they’re a little bit co-dependent, and maybe this Veteran is otherwise…a fairly high-functioning Veteran, maybe someone that they really like and they[‘re] kind of like, ‘Well, you know you should stop. We can help you when you’re ready’ or they just basically kind of ignore it and slide it under the carpet…"*

One provider pointed to avoidance of the issue in primary care.

*"And I think a lot of the primary care providers tend to sort of see that whole area [hazardous substance use and SUD] as just a big pain in the you know what. Just a big headache. So would rather not ask Veterans [about their substance use]."*

Another provider talked about the power of active listing saying: *“My big skill in the world is listening.”*

*"When you listen… you can start reading between the lines and knowing for a fact that there’s a tight association between trauma and substance abuse or at least alcoholism and even more so in the Veteran population, where you’re literally taught to drink in the military, taught to smoke… That’s just one of the things that I would listen for and then start asking some questions about and eventually the truth would reveal itself."*

### Theme 4: Patient factors and how they self-identify may impact identification of women Veterans with hazardous substance use

Providers reported substantial variation in the way Veterans perceive themselves and assign themselves an identity of Veteran or non-Veteran. This self-perception is separate from technical Veteran status/VHA eligibility. For example, some eligible women Veterans with SUD (i.e., have served in the military, have Veteran status, and SUD) may qualify to receive SUD care through the VHA, but do not self-identify as Veterans.

*"…even women [Veterans with SUD] who had successful military careers and so on and so forth, as we all know that we’ve been researching these issues for so many decades now, thank you, is that a lot of women won’t say yes to that question [are you a Veteran] anyway."*

Further, many eligible women Veterans face life circumstances or environmental barriers that prevent them from being identified for and/or receiving SUD care in the VHA, for example, homelessness or at-risk living situations:

*"Now we know that homeless women Veterans are not necessarily homeless in that sort of like stereotypical under the bridge, under the freeway bridge with a cart kind of circumstance, but they’re out there somewhere in these really at-risk living situations and a lot of times I think that those environmental factors prevent them from ever getting into us for [SUD] care."*

Providers advocated a need for an identification approach for women Veterans that is inclusive of women Veterans with hazardous substance use and SUD who may not currently be receiving their care in VHA.

### Theme 5: VHA eligibility and implications for hazardous substance use identification

Some women with SUD who have served in the military may not be eligible for Veteran benefits because they do not meet various requirements (e.g., have a less than honorable discharge). It was noted that to assess military history, providers at touchpoints outside of the VHA are reframing their lines of questioning:

*"A lot of them end up getting sort of sucked up into the legal system and may in fact be in jails. One of the things that everybody that’s doing the jail work and the prison work is really learning is we’re not asking the women, “Are you Veterans?” We’re asking, “Did you ever serve in the military,” right? Because they know they’re not Veterans."*

Other providers noticed that women with military service often do not self-identify as Veterans because they see benefits status as the criteria for being a Veteran.

*"...women who have attempted to get their VHA benefits and have been told ‘you’re ineligible [for VHA benefits],’ they don’t think of themselves as Veterans at all, but if you ask them ‘did you serve in the military,’ you get a whole different set of replies and you start identifying a number of women [with hazardous substance use who need care] that way."*

One provider expressed concern that when screening for hazardous substance use and SUD outside of the VHA, some women may self-identify as Veterans but not actually be eligible for care in the VHA.

*"A lot of those women [women with hazardous substance use or SUD who self-identify as a Veteran due to their history of military service] were not able to complete a full tour of duty in the military and so they’re not eligible [for Veteran benefits]…"*

## Discussion

Hazardous substance use has been linked with many negative health outcomes, [29] and SUD is a chronic and life-threatening health condition [7, 30]. Because hazardous substance use indicates risk of harm and symptoms can present as “red flags,” [7] providers who identify hazardous substance use may provide their women Veteran patients with early intervention by way of referral and appropriate treatment. Although there are evidence-based treatments for SUD, patients lack access to these treatments when their hazardous substance use goes unrecognized and potential SUD goes undiagnosed [31]. A history of punitive policies, provider bias, and stigma have hampered case-finding in the past, which creates a perfect storm scenario whereby screening and proper identification have the real potential to improve access to specialty SUD care for those patients most likely to benefit from it.

However, implementation of a screening program, even when referral to specialty care is a feasible care pathway is likely not adequate. Providers in our study noted that use of screening tools to identify hazardous substance use or SUD outside of trusting provider-patient relationships may not elicit the true extent of a patient’s hazardous substance use. This is particularly true for women Veterans given the heightened stigma and potential consequences they may face, particularly during pregnancy, [8] if they divulge hazardous substance use. Stigma around substance use and use disorders impacts women more than men with the same diagnosis [32]. In the absence of trust, a patient may be less likely to self-report hazardous substance use if they see no pathway to a benefit (e.g., treatment without stigma or perceived negative consequences). Providers indicated that screening for hazardous substance use must take place within a trusting patient-provider relationship given patients’ fear of negative consequences.

In this secondary analysis to understand the different ways women Veterans with hazardous substance use are identified, providers mentioned that screening tools, including screening questionnaires like the AUDIT-C, are some of the primary means by which identifications and referrals are made. Providers believed that identifying women Veterans with hazardous substance use is currently hindered by limitations of current screening methodologies; and screening tools are not typically repeated at frequent enough intervals to capture critical changes in status as a response to related care or intervention. Our results and the supporting literature highlight the need to tailor hazardous substance use and SUD identification methodologies, particularly for groups such as women who face increased stigma related to their hazardous substance use [33–35]. More patient-centered screening practices can foster the candid conversations between patients and providers that ultimately yield critical information to guide appropriate referral, treatment planning, and intervention. However, tailoring screening tools alone is not sufficient: If patients fear repercussions from being honest about the extent of their hazardous substance use, the screening and identification process will still fall short. Fundamentally, screening via questionnaire or interpersonal patient-provider dialogue relies on the patient feeling safe enough to self-report hazardous substance use.

Providers also noted several factors hindering their own ability to identify hazardous substance use in their women Veteran patients, including their perceived infrequent exposure to this population, providers’ stated inclination to dismiss hazardous substance use in high functioning patients, and their reluctance to address hazardous substance use questions with patients. These findings may be critically important to understanding provider’s self-identified reluctance to identify their women Veteran patients as having hazardous substance use, which would require referral for further evaluation and result potential SUD diagnosis, when viewed in the context of a seminal systematic review looking at provider stigma toward patients with SUD [4]. The van Boekel article found that providers generally displayed negative attitudes toward patients with SUD and viewed them as being “difficult to treat” because providers perceived these patients as more violent, manipulative, and unmotivated than patients without SUD [4]. This aligns with our finding in Theme 3 that some providers consider dealing with hazardous substance use a burden they would rather avoid, leading them to not ask questions about use altogether. Interestingly, it may also explain why providers who expressed empathy for their women Veteran patients who they may perceive as high functioning and/or acknowledged their higher likelihood of trauma and dismissed/normalized potentially hazardous use.

Our findings are also consistent with literature indicating that provider biases against women with hazardous substance use and SUD leads to under identification and avoidance of discussing hazardous substance use with women patients. Individual provider biases may contribute to a system-level inability to identify women Veterans with hazardous substance use. Addressing this system shortfall may require education and training at the provider level [11]. Gender biases impact the patient-provider relationship, which can affect access to appropriate healthcare as well as health outcomes [36]. Interventions at a system level, including provider training about gender sensitivity in healthcare delivery, [36] are a tool to improve care access by ensuring women Veterans with hazardous substance use are appropriately identified and referred for SUD evaluation, diagnosis, and treatment.

How patients self-identify their Veteran status plays a role in the ability to identify women Veterans with hazardous substance use Hazardous substance use has more significant consequences for women, including higher rates of untreated mental health issues as well as higher risk of incarceration, domestic violence, and homelessness [11]. Additionally, women Veterans are more likely to have trauma histories than non-Veterans, [13] and are at a higher risk of experiencing military sexual trauma (MST) than their men counterparts.[37] Women Veterans who have survived MST are less likely to seek treatment through the VHA, despite their eligibility, partly due to feelings of institutional betrayal – i.e., poor institutional response to MST may lead survivors to seek healthcare in civilian settings as opposed to identifying as a Veteran for the purposes of receiving healthcare through the VHA [37]. This may contribute to our findings that some providers believe they encounter few women Veterans with hazardous substance use. This is surprising considering the high prevalence of trauma history, a risk factor for hazardous substance use and SUD, among women Veteran patients. Patients are multifaceted with self-assigned identities; to succeed, hazardous substance use screening and identification efforts must consider and accommodate how patients view themselves.

A major implication for Veteran health is that there is a need to designate solutions and responsibility for vulnerable persons that are identified as having hazardous substance use issues, but who are not diagnosed with SUD or receiving care in VHA (e.g., due to issues such as lack of VHA benefits). In the absence of VHA access, it is an open question whether and where they can receive support and services whose responsibility is it to connect them with such resources.

## Limitations:

We conducted the qualitative interviews for this study in 2014 and providers’ perceptions may have changed in the subsequent years. However, given the critical gap in the current literature, these findings can nonetheless help us advance the conceptual thinking around screening and hazardous substance use identification. Although we strategically targeted providers since screening and identification are systems-level issues, expanding this work to include more of the patient voice is warranted [37]. Future work to improve hazardous substance use screening and identification should further consider Veteran perspectives.

## Conclusions

The process of identifying and referring women Veterans with hazardous substance use to appropriate care should focus on building trust in patient-provider relationships, creating system-level solutions for provider biases relating to gender and SUD, and accommodating patient identities. There are women Veterans who would be eligible for SUD treatment and other care through VHA, but who are currently not in the VHA system. To fully address the challenges of identifying women Veterans with hazardous substance use, screening and identification efforts will need to extend beyond the VHA walls. Through coordinating efforts with social services and justice systems, facilitating referrals may help capture women who are eligible for care in VHA but who have, for various reasons, not identified as Veterans.

## Data Availability

The datasets during and/or analyzed during the current study available from the corresponding author on reasonable request.

## Abbreviations

VHA: Veterans Health Administration
VA: Veterans Affairs
SUD: substance use disorder/s
PTSD: post-traumatic stress disorder
MST: military sexual trauma (MST)

## References

[1] Ram A, Chisolm MS (2016). The Time is Now: Improving Substance Abuse Training in Medical Schools. Acad Psychiatry.

[2] Upshur CC, Luckmann RS, Savageau JA (2006). Primary care provider concerns about management of chronic pain in community clinic populations. J Gen Intern Med.

[3] Szalavitz M, Rigg KK, Wakeman SE (2021). Drug dependence is not addiction—and it matters. Ann Med.

[4] Van Boekel LC, Brouwers EP, Van Weeghel J, Garretsen HF (2013). Stigma among health professionals towards patients with substance use disorders and its consequences for healthcare delivery: systematic review. Drug Alcohol Depend.

[5] Nguyen  T, Buxton  JA (2021). Pathways between COVID-19 public health responses and increasing overdose risks: a rapid review and conceptual framework. International Journal of Drug Policy.

[6] Martirosyan L, Arah OA, Haaijer-Ruskamp FM, Braspenning J, Denig P (2010). Methods to identify the target population: implications for prescribing quality indicators. BMC Health Serv Res.

[7] Saunders JB, Latt NC (2021). Diagnostic Definitions and Classification of Substance Use Disorders.

[8] Stone R (2015). Pregnant women and substance use: fear, stigma, and barriers to care. Health & Justice.

[9] Laramee P, Leonard S, Buchanan-Hughes A, Warnakula S, Daeppen JB, Rehm J (2015). Risk of All-Cause Mortality in Alcohol-Dependent Individuals: A Systematic Literature Review and Meta-Analysis. EBioMedicine.

[10] Jane-Llopis E, Matytsina I (2006). Mental health and alcohol, drugs and tobacco: a review of the comorbidity between mental disorders and the use of alcohol, tobacco and illicit drugs. Drug Alcohol Rev.

[11] Goodman DJ, Wolff KB (2013). Screening for substance abuse in women’s health: a public health imperative. J Midwifery Womens Health.

[12] Cucciare MA, Simpson T, Hoggatt KJ, Gifford E, Timko C (2013). Substance use among women veterans: epidemiology to evidence-based treatment. J Addict Dis.

[13] Najavits LM, Weiss RD, Shaw SR (1997). The link between substance abuse and posttraumatic stress disorder in women. A research review. Am J Addict.

[14] Creech SK, Borsari B (2014). Alcohol use, military sexual trauma, expectancies, and coping skills in women veterans presenting to primary care. Addict Behav.

[15] Myrick H, Brady K (2003). Current review of the comorbidity of affective, anxiety, and substance use disorders. Curr Opin Psychiatry.

[16] Chapman SLC, Wu L-T (2014). Suicide and substance use among female veterans: A need for research. Drug Alcohol Depend.

[17] Oyserman D, Fryberg SA, Yoder N (2007). Identity-based motivation and health. J Personal Soc Psychol.

[18] SAMHSA (2009). Substance abuse treatment: Addressing the specific needs of women. HHS publication.

[19] Campbell CI, Alexander JA (2005). Health services for women in outpatient substance abuse treatment. Health Serv Res.

[20] Greenfield SF, Back SE, Lawson K, Brady KT (2010). Substance abuse in women. Psychiatric Clin.

[21] Davis TM, Bush KR, Kivlahan DR, Dobie DJ, Bradley KA (2003). Screening for substance abuse and psychiatric disorders among women patients in a VA Health Care System. Psychiatric Serv.

[22] Lundin A, Hallgren M, Balliu N, Forsell Y (2015). The use of alcohol use disorders identification test (AUDIT) in detecting alcohol use disorder and risk drinking in the general population: validation of AUDIT using schedules for clinical assessment in neuropsychiatry. Alcoholism: Clin Experimental Res.

[23] Rumpf H-J, Hapke U, Meyer C, John U (2002). Screening for alcohol use disorders and at-risk drinking in the general population: psychometric performance of three questionnaires. Alcohol Alcohol.

[24] Dawson   DA, Grant  BF,  Stinson  FS,  Zhou  Y (2005). Effectiveness of the derived Alcohol Use Disorders Identification Test (AUDIT-C) in screening for alcohol use disorders and risk drinking in the US general population. Alcohol Clin Exp Res.

[25] Lee  JH, Jung   KY, Choi YH  (2018). Screening test for at-risk drinking: development of new abbreviated version of Alcohol Use Disorder Identification Test for young and middle-aged adults.. Emerg Med Int.

[26] Biernacki P, Waldorf D (1981). Snowball sampling: Problems and techniques of chain referral sampling. Sociol methods Res.

[27] Coyne IT (1997). Sampling in qualitative research. Purposeful and theoretical sampling; merging or clear boundaries?. J Adv Nurs.

[28] Giannitrapani KF, Huynh AK, Schweizer CA, Hamilton AB, Hoggatt KJ (2018). Patient-centered substance use disorder treatment for women Veterans. J Military Veteran Family Health.

[29] Owens GP, Held P, Blackburn L, Auerbach JS, Clark AA, Herrera CJ (2014). Differences in relationship conflict, attachment, and depression in treatment-seeking veterans with hazardous substance use, PTSD, or PTSD and hazardous substance use. J interpers Violence.

[30] ASAM (2019). Definition of Addiction American Society of Addiction Medicine.

[31] Goodman D, Whalen B, Hodder LC (2019). It’s time to support, rather than punish, pregnant women with substance use disorder. JAMA Netw open.

[32] Stringer KL, Baker EH (2018). Stigma as a barrier to substance abuse treatment among those with unmet need: an analysis of parenthood and marital status. J Fam Issues.

[33] Hoggatt KJ, Simpson T, Schweizer CA, Drexler K, Yano EM (2018). Identifying women veterans with unhealthy alcohol use using gender-tailored screening. Am J addictions.

[34] Hoggatt  KJ,  Harris  AH,  Washington  DL,  Williams  EC (2021). Prevalence of substance use and substance-related disorders among US Veterans Health Administration patients. Drug Alcohol Depend.

[35] Williams EC, Lapham GT, Rubinsky AD, Chavez LJ, Berger D, Bradley KA (2017). Influence of a targeted performance measure for brief intervention on gender differences in receipt of brief intervention among patients with unhealthy alcohol use in the Veterans Health Administration. J Subst Abuse Treat.

[36] Govender V, Penn-Kekana L (2008). Gender biases and discrimination: a review of health care interpersonal interactions. Glob Public Health..

[37] Monteith   LL, Holliday   R, Schneider   AL, Miller  CN,  Bahraini  NH, Forster   JE (2021). Institutional betrayal and help-seeking among women survivors of military sexual trauma. Psychological Trauma: Theory, Research, Practice, and Policy.

